# Optimizing microbiome sequencing for small intestinal aspirates: validation of novel techniques through the REIMAGINE study

**DOI:** 10.1186/s12866-019-1617-1

**Published:** 2019-11-01

**Authors:** Gabriela Guimaraes Sousa Leite, Walter Morales, Stacy Weitsman, Shreya Celly, Gonzalo Parodi, Ruchi Mathur, Rashin Sedighi, Gillian M. Barlow, Ali Rezaie, Mark Pimentel

**Affiliations:** 1 Medically Associated Science and Technology (MAST) Program, Cedars-Sinai Medical Center, Los Angeles, CA USA; 2 Division of Endocrinology, Diabetes, and Metabolism, Cedars-Sinai Medical Center, Los Angeles, CA USA; 3 Division of Digestive and Liver Diseases, Cedars-Sinai Medical Center, Los Angeles, CA USA

**Keywords:** Small intestine, Microbiome, Mucus layer, Methodology optimization, Microbial culture, 16S rRNA gene sequencing

## Abstract

**Background:**

The human small intestine plays a central role in the processes of digestion and nutrient absorption. However, characterizations of the human gut microbiome have largely relied on stool samples, and the associated methodologies are ill-suited for the viscosity and low microbial biomass of small intestine samples. As part of the REIMAGINE study to examine the specific roles of the small bowel microbiome in human health and disease, this study aimed to develop and validate methodologies to optimize microbial analysis of the small intestine.

**Results:**

Subjects undergoing esophagogastroduodenoscopy without colon preparation for standard of care were prospectively recruited, and ~ 2 ml samples of luminal fluid were obtained from the duodenum using a custom sterile aspiration catheter. Samples of duodenal aspirates were either untreated (DA-U, *N* = 127) or pretreated with dithiothreitol (DA-DTT, *N* = 101), then cultured on MacConkey agar for quantitation of aerobic gram-negative bacteria, typically from the class Gammaproteobacteria, and on blood agar for quantitation of anaerobic microorganisms. DA-DTT exhibited 2.86-fold greater anaerobic bacterial counts compared to DA-U (*P* = 0.0101), but were not statistically different on MacConkey agar.

DNA isolation from DA-U (*N* = 112) and DA-DTT (*N* = 43) samples and library preparation for 16S rRNA gene sequencing were also performed using modified protocols. DA-DTT samples exhibited 3.81-fold higher DNA concentrations (*P* = 0.0014) and 4.18-fold higher 16S library concentrations (*P* < 0.0001) then DA-U samples. 16S rRNA gene sequencing revealed increases in the detected relative abundances of obligate and facultative anaerobes in DA-DTT samples, including increases in the genera *Clostridium* (false discovery rate (FDR) *P* = 4.38E-6), *Enterococcus* (FDR *P* = 2.57E-8), *Fusobacterium* (FDR *P* = 0.02) and *Bacteroides* (FDR *P* = 5.43E-9). Detected levels of Gram-negative enteropathogens from the phylum *Proteobacteria*, such as *Klebsiella* (FDR *P* = 2.73E-6) and *Providencia* (FDR *P* < 0.0001) (family *Enterobacteriaceae*) and *Pseudomona*s (family *Pseudomonadaceae*) (FDR *P* = 0.04), were also increased in DA-DTT samples.

**Conclusions:**

This study validates novel DTT-based methodology which optimizes microbial culture and 16S rRNA gene sequencing for the study of the small bowel microbiome. The microbial analyses indicate increased isolation of facultative and obligate anaerobes from the mucus layer using these novel techniques.

## Background

The Human Microbiome Project [1] was a groundbreaking introduction to the understanding of the microbiome of the human body. In this effort, many areas of the human microbiome were sampled and characterized, such as the mouth, nose, integument and vaginal tract [2]. The gastrointestinal tract was specifically characterized using stool as an easily available surrogate. Although stool sequencing may reveal the microbial signature of the distal colon, it is well known that stool does not adequately represent the entire gastrointestinal tract, given the multiple environments that exist as one travels from the stomach to the small bowel and then the colon [3]. For example, conditions such as acidity and transit time vary tremendously along the intestine with likely significant effects on microbes by area of exam [3].

In contrast to the colon, the small intestine, which is divided into the duodenum, jejunum and ileum, is of central importance to digestion and nutrient absorption. Of these, the duodenum has great importance as the site of convergence of chyme from the stomach, enzymes from the pancreas and bile salts from the gall bladder. Clearly, characterizing the microbial populations of the small intestine is of central importance, but efforts to date have been hampered both by the difficulty of obtaining samples, and by challenges associated with adapting sample processing for DNA isolation techniques that were designed for stool.

The REIMAGINE (Revealing the Entire Intestinal Microbiota and its Associations with the Genetic, Immunologic, and Neuroendocrine Ecosystem) study is a large-scale initiative to examine the specific importance of the small bowel microbiome in human health and disease (https://www.cedars-sinai.org/programs/digestive-liver-diseases/clinical/small-bowel-diseases-nutrition/clinical-trials/small-intestinal-sampling-study.html). However, in order to adequately assess the small bowel, techniques need to be assessed and optimized for this location of the intestinal tract. In addition to maintaining sterility and preventing cross-contamination with oral and stomach microbes, particular challenges associated with processing and isolation of DNA from small intestinal samples include the viscosity, small sample volumes, and low microbial biomass. Recently, techniques and adaptations have been described aimed at optimizing DNA sequencing from low-biomass [4], but not high viscosity samples. Viscosity of the small bowel mucous could impede or affect DNA isolation. One possible remedy would be to reduce viscosity prior to DNA isolation by treating the aspirates with dithiothreitol (DTT), which is commonly used to reduce the disulfide bonds between cysteine residues of proteins and can also reduce the disulfide bonds linking mucin subunits in mucus, improving bacterial recovery and DNA extraction methods [5]. DTT has previously been used to liquefy sputum samples for DNA extraction [5].

In this study we attempt to develop and validate methodologies to optimize microbial analysis of the small intestine as part of the REIMAGINE study. Optimization included utilization of a new catheter technique for aspiration, steps to improve DNA recovery using DTT, and a new DNA library preparation technique in comparison to conventional DNA isolation and sequencing.

### Results

#### Samples and treatment

**Fig. 1:**
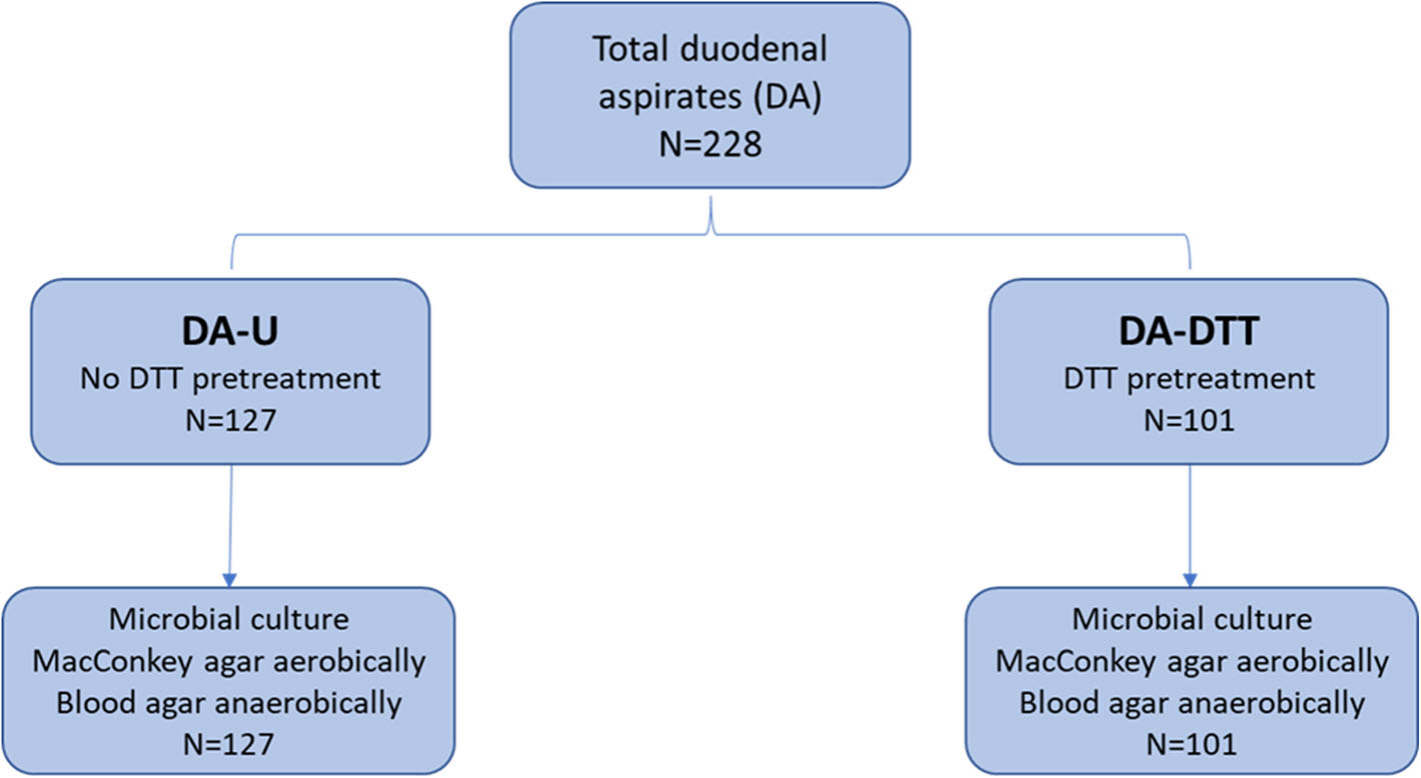
Workflow for pretreatment and microbial culture, including the number of subjects in each group

A total of 228 subjects had DA samples collected and analyzed as shown in Fig. 1. Of these, 127 DA were not pretreated with DTT prior to microbial culture (DA-U, the untreated group), and 101 were pretreated with DTT prior to microbial culture (the DA-DTT group).

#### DTT effect on microbial cultures

No growth was observed in MacConkey agar plated with 1x DTT only (negative control). The CFU on MacConkey agar obtained from DA-U subjects ranged from 0 to 240 × 10^3^ CFU/mL (Mean = 10.6 × 10^3^ CFU/mL, Median = 0 CFU/mL, 25th percentile = 0 CFU/mL and 75th percentile = 0 CFU/mL). In the DA-DTT group, the CFU on MacConkey agar ranged from 0 to 1035 × 10^3^ CFU/mL (Mean = 28.22 × 10^3^ CFU/mL, Med = 0 CFU/mL, 25th percentile = 0 CFU/mL and 75th percentile = 2.5 × 10^3^ CFU/mL) (Additional file 1). For the purposes of statistical analysis only, no growth was designated as 1 bacterial CFU/mL of aspirate. DA-DTT exhibited 2.6-fold greater bacterial colonies on MacConkey agar when compared to DA-U, but the *p*-value did not reach statistical significance (*P* = 0.14).

No growth was observed on blood agar cultured with 1x DTT only (negative control). The CFU on blood agar obtained from DA-U subjects ranged from 0 to 800 × 10^3^ CFU/mL (Mean = 31.3 × 10^3^ CFU/mL, Median = 0 CFU/mL, 25th percentile = 0 CFU/mL and 75th percentile = 20 × 10^3^ CFU/mL) (Additional file 1). On blood agar, DA-DTT exhibited 2.86-fold greater anaerobic bacterial colonies when compared to DA-U (*P* = 0.0101). For the purposes of statistical analysis only, no growth was designated as 1 bacterial CFU/mL of aspirate. In the DA-DTT group, CFU on blood agar ranged from 0 to 2070 × 10^3^ CFU/ml (Mean = 89.58 × 10^3^ CFU/mL, Med = 6x10^3^CFU/mL, 25th percentile = 0 CFU/mL and 75th percentile = 98.5 × 10^3^ CFU/mL).

#### Immediate post aspiration DTT improves DNA extraction and 16S metagenomic library preparation for DA

**Fig. 2:**
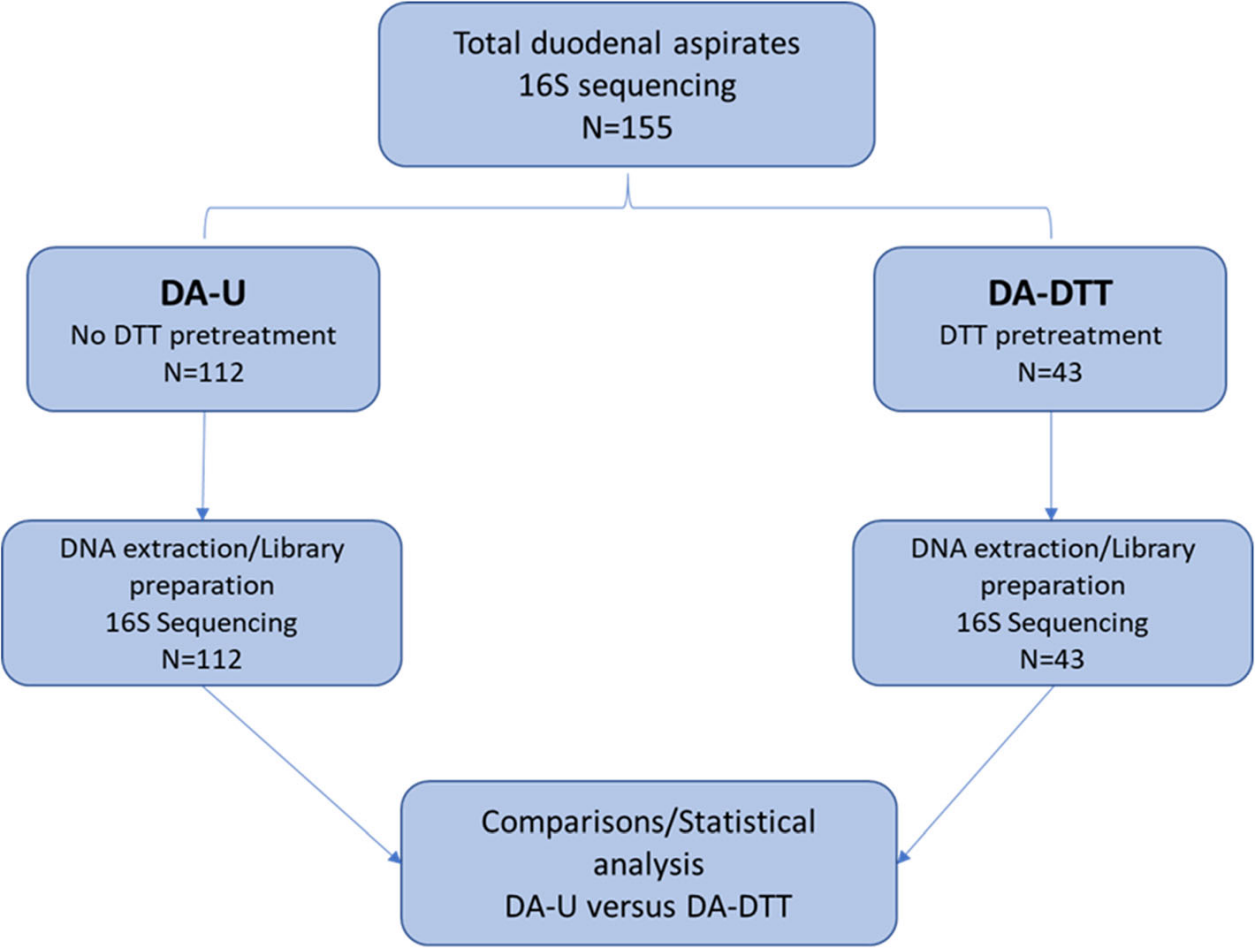
Workflow for DNA extraction and 16S rRNA gene sequencing of duodenal aspirate (DA) samples, including the number of subjects in each group

A total of 155 subjects had their DA samples sequenced and analyzed as shown in Fig. 2 (Additional file 1). The concentrations of DNAs obtained from negative controls (DTT only) were undetectable. The concentrations of DNAs obtained from DA-U subjects ranged from undetectable levels (lower than 10 pg/μL) (*n* = 18) to 24.6 ng/μL (Med = 0.0908 ng/μL, 25th percentile = 0.02365 ng/μL and 75th percentile = 0.6875 ng/μL) (Additional file 1). Treatment with DTT improved DNA isolation. In the DA-DTT group, DNA concentrations ranged from undetectable levels (*n* = 3) to 68.8 ng/μL (Med = 0.346 ng/μL, 25th percentile = 0.0906 ng/μL and 75th percentile = 1.91 ng/μL) and were 3.81-fold higher than those from DA-U (Mann Whitney *P* = 0.0014).

The concentrations of the final 16S libraries amplified from negative controls were undetectable. The concentrations of the final 16S libraries amplified from DA-U samples (i.e. those for which DTT was added only for the removal of the All Protect reagent) ranged from 0.14 ng/μL to 136 ng/μL (median = 21.8 ng/μL, 25th percentile = 5.1 ng/μL and 75th percentile = 69.8 ng/μL) and correlated with the initial DNA concentrations (Spearman r = 0.316, P = 0.001) (Additional file 1).

**Fig. 3:**
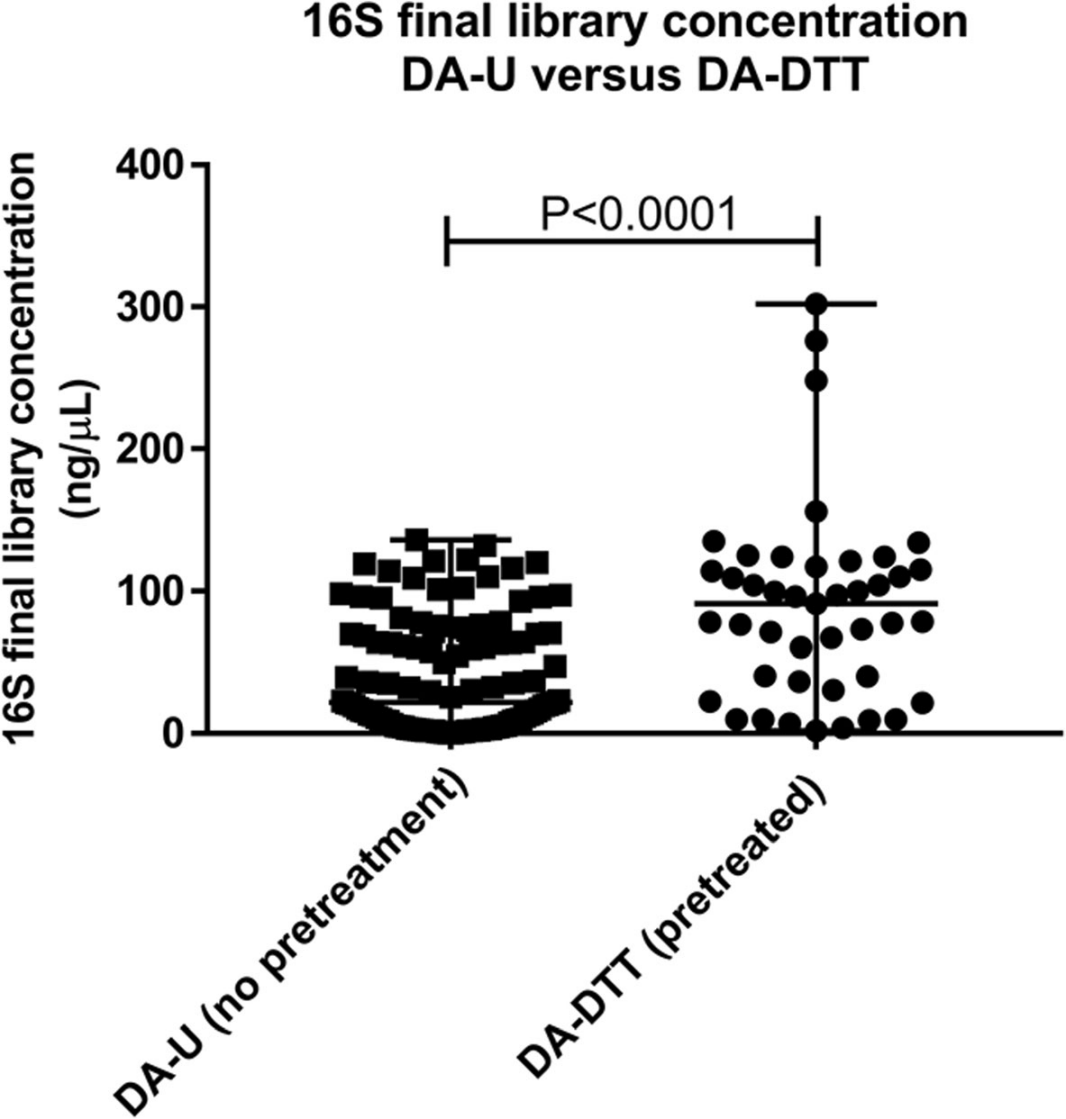
Final quantification of 16S libraries from DA-U (*N* = 112) and DA-DTT (*N* = 43) samples after 35 PCR cycles. The Mann-Whitney test was used to compare the median value of groups

The concentrations of the final 16S libraries amplified from DA-DTT samples (i.e. those for which DTT was added both before microbial culture and for removal of the All Protect reagent) were 4.18x higher than those of libraries from DA-U samples (*P* < 0.0001) (see Fig. 3). The library concentrations ranged from 1.69 ng/μL to 302 ng/μL (Med = 91.2 ng/μL, 25th percentile = 36.6 ng/μL and 75th percentile = 117 ng/μL) and correlated with the initial DNA concentrations (Spearman r = 0.443, *P* = 0.003) (Additional file 1).

#### Sequencing results

All samples had at least 9000 sequences and no exclusions were performed. A total of 112 DA-U and 43 DA-DTT samples were sequenced. The difference in average library sizes between the groups was less than 2-fold (Additional file 2). Predictions for significant differentially abundant Operational Taxonomic Units (OTUs) were performed following recommendations from McMurdie and Holmes [6], and from Weiss et al. [7], used when the average library size for each group is approximately equal and/or the fold difference between groups is not high (> 2-3x on average) (Additional file 1).

Considering observations regarding contamination of DNA extraction kits with traces of bacterial DNA [8],16S sequencing was also performed on negative control samples (DTT only). Less than 0.03% of the total sequences generated in each MiSeq run was assigned to negative control samples, 4433 sequences on average. 27.63% of the sequences assigned to negative controls were identified as bacterial DNA, mostly belong to the *Pseudomonas* genus OTU 646549 (63.5%), and *Bacteroides* genus OTUs 1,749,079, 193,591 and 359,538 (12%).

All OTUs observed in negative controls were also detected in both groups analyzed, DA-U and DA-DTT. The OTUs assigned to *Bacteroides* genus observed in negative controls represented less than 3% of all OTUs assigned to this same genus in DA-U and DA-DTT, thus these OTUs were not excluded during downstream analysis. The OTU assigned to *Pseudomonas* genus (646549) observed in negative controls represented 67% of the OTUs assigned to this same genus in DA-U and DA-DTT, and considering the high risk of bias during analysis the OTU 646549 was excluded during comparisons between DA-U and DA-DTT groups (Additional file 1).

#### DTT increases the detected relative abundance of anaerobic bacteria in DA

**Fig. 4:**
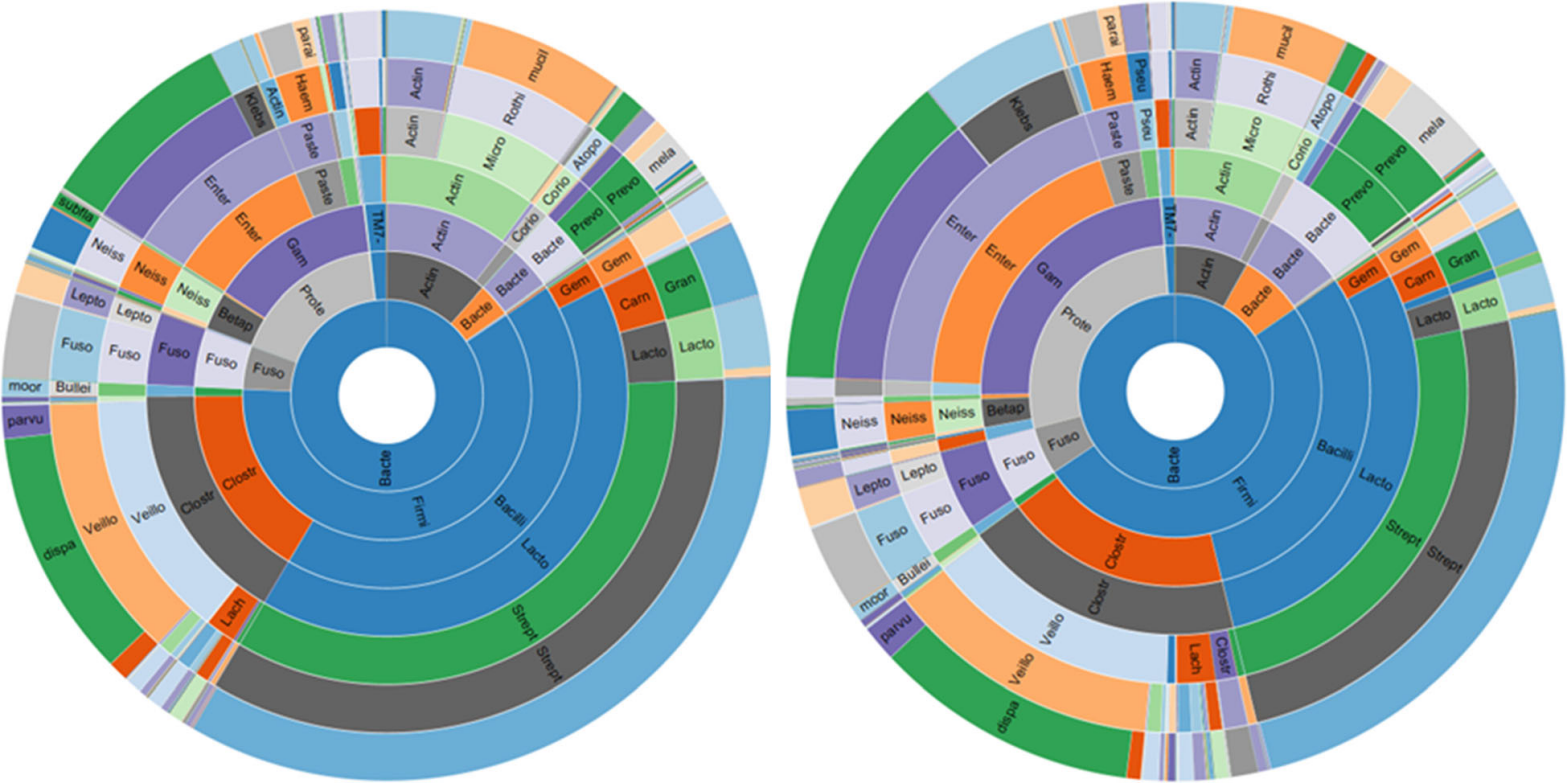
Sunburst representation of the overall distribution of the small intestinal microbiome as determined by 16S rRNA sequencing. On the left: Relative microbial abundance detected in DA-U (no pretreatment, *N* = 112). On the right: Relative microbial abundance detected in DA-DTT (pretreatment with DTT, *N* = 43)

The main two dominant phyla observed in DA-DTT and DA-U were *Firmicutes* and *Proteobacteria*, followed by smaller proportions of *Actinobacteria, Fusobacteria, Bacteroidetes* and *TM7* (Fig. 4, Table 1). After pretreatment with DTT, DA showed increased relative abundance of the phyla *Proteobacteria* (FC = 6.22, FDR *P* = 7.71E-7), *Bacteroidetes* (FC = 2.19, FDR *P* = 0.03) and *Fusobacteria* (FC = 1.96, FDR P = 0.03), when compared to DA-U (Table 1). There were also smaller changes in the relative abundances of Actinobacteria and TM7 that did not reach significance (Table 1).

**Table 1:** Differential abundance of the top six phyla in DA-DTT versus DA-U

	DA-DTT (n = 43) versus DA-U (*n* = 112)				
Taxonomy	Average Relative abundance % DA-DTT^a^	Average Relative abundance % DA-U^a^	Fold Change (calculated from the GLM)^b^	*P*-value	FDR *P*-value
Firmicutes	49.3	62.25	1.05	0.65	0.70
Proteobacteria	28.97	14.8	6.22	**1.4E-7**	**7.71E-7**
Actinobacteria	8.91	12.02	−1.23	0.21	0.42
Fusobacteria	5.36	3.93	1.96	**0.01**	**0.03**
Bacteroidetes	6.16	4.63	2.19	**0.01**	**0.03**
TM7	1.17	1.86	−1.34	0.32	0.48

*P*-value< 0.05 and FDR *P*-value< 0.05 are shown in bold. ^a^The relative abundances were calculated from the original counts (number of sequences in the OTU table). ^b^Fold changes were calculated from the GLM, which corrects for differences in library size between the samples and the effects of confounding factors. It is therefore not possible to derive these fold changes from the original counts (number of sequences in the OTU table) by simple algebraic calculations

Although no changes were seen in the detected relative abundances of *Clostridia* and *Bacilli*, the two main classes from the phylum *Firmicutes*, in DA-DTT vs. DA-U, specific increases were observed in families from both of these classes. Specifically, DA-DTT exhibited increased detected relative abundances of the family *Clostridiaceae* (FC = 5.10, FDR P < 0.0001) and genus *Clostridium* (FC = 4.06, FDR P = 4.38E-6), which are Gram-positive obligate anaerobes, and of the family *Enterococcaceae* (FC = 76.22, FDR *P* = 2.62E-11) and genus *Enterococcus* (FC = 42.18, FDR P = 2.57E-8), which are Gram-positive facultative anaerobic lactic acid bacteria (Table 2, Fig. 4).

**Table 2:** Differential abundance of anaerobic bacteria in DA-DTT versus DA-U

	DA-DTT (*n* = 43) vs. DA-U (*n* = 112)				
Taxonomy	Average Relative abundance % DA-DTT^a^	Average Relative abundance % DA-U^a^	Fold Change (calculated from the GLM)^b^	*P*-value	FDR *P*-value
p_Firmicutes, c_Clostridia, f_Clostridiaceae, g_Clostridium	0.032	0.024	4.06	**1.22E-6**	**4.38E-6**
p_Firmicutes, c_Bacilli, f_Enterococcaceae, g_Enterococcus	0.661	0.009	42.18	**5.57E-9**	**2.57E-8**
p_Fusobacteria, c_Fusobacteriia, f_Fusobacteriaceae, g_Fusobacterium	3.625	2.471	2.29	**0.01**	**0.02**
p_Bacteroidetes, c_Bacteroidia, f_Bacteroidaceae, g_Bacteroides	0.626	0.073	28.08	**1.08E-9**	**5.43E-9**

*P*-value< 0.05 and FDR *P*-value< 0.05 are shown in bold. ^a^The relative abundances were calculated from the original counts (number of sequences in the OTU table). ^b^Fold changes were calculated from the GLM, which corrects for differences in library size between the samples and the effects of confounding factors. It is therefore not possible to derive these fold changes from the original counts (number of sequences in the OTU table) by simple algebraic calculations

The detected relative abundances of several obligate anaerobic bacteria were increased in DA-DTT vs. DA-U, including *Fusobacterium* (phylum *Fusobacteria*), which are Gram-negative bacilli (FC = 2.29, FDR *P* = 0.02), and *Bacteroides* (phylum *Bacteroidetes*) (FC = 28.08, FDR *P* = 5.43E-9) (Table 2, Fig. 4).

#### Pretreatment with DTT increased the detected relative abundance of gram-negative enteropathogens from the phylum Proteobacteria

The relative abundance of the phylum *Proteobacteria*, a major phylum of Gram-negative bacteria, detected in DA-DTT was increased compared to that detected in DA-U (FC = 6.22, FDR P = 7.71E-7). The detected relative abundances of three of the five most important classes from this phylum were significantly increased in DA-DTT compared to DA-U - class *Gammaproteobacteria*, which comprises several enteropathogens (FC = 8.44, FDR *P* = 4.25E-8) [9], class *Alphaproteobacteria*, which includes mainly phototrophic bacteria (FC = 7.94, FDR *P* = 2.60E-8), and class *Deltaproteobacteria*, which includes sulfate- and sulfur-reducing bacteria (FC = 6.35, FDR *P* = 9.7E-5) (Table 3). Smaller changes in classes *Betaproteobacteria* and *Epsilonproteobacteria* did not reach significance (Table 3).

**Table 3:** Differential abundance of members of the phylum Proteobacteria in DA-DTT versus DA-U

	DA-DTT (*n* = 43) vs. DA-U (*n* = 112)				
Taxonomy	Average relative abundance % DA-DTT^a^	Average relative abundance % DA-U^a^	Fold Change (calculated from the GLM)^b^	*P*-value	FDR *P*-value
p_Proteobacteria, c_Gammaproteobacteria	23.823	10.492	8.44	**8.3E-9**	**4.25E-8**
p_Proteobacteria, c_Alphaproteobacteria	1.294	0.145	7.94	**4.05E-9**	**2.60E-8**
p_Proteobacteria, c_Deltaproteobacteria	0.008	0.001	6.35	**3.08E-5**	**9.70E-5**
p_Proteobacteria, c_Betaproteobacteria	3.569	4.029	−1.26	0.41	0.56
p_Proteobacteria, c_Epsilonproteobacteria	0.281	0.167	1.74	0.14	0.29

*P*-value< 0.05 and FDR *P*-value< 0.05 are shown in bold. ^a^The relative abundances were calculated from the original counts (number of sequences in the OTU table). ^b^Fold changes were calculated from the GLM, which corrects for differences in library size between the samples and the effects of confounding factors. It is therefore not possible to derive these fold changes from the original counts (number of sequences in the OTU table) by simple algebraic calculations

The increase in the detected relative abundance of *Gammaproteobacteria* in DA-DTT was partially driven by higher detected relative abundances of *Enterobacteriaceae* family members (FC = 5.46, FDR *P* = 1.47E-3), including important enteropathogens and pathogens that cause infection in several parts of the human body, such as *Klebsiella* and *Providencia* (see Table 4) [10]. The detected relative abundances of other members of the class *Gammaproteobacteria* were also increased in DA-DTT vs. DA-U, including the family *Aeromonadaceae* (FC = 63.61, FDR *P* = 1.18E-13) and the genus *Pseudomonas* (family *Pseudomonadaceae*) (FC = 2.65, FDR *P* = 0.04).

**Table 4:** Differential abundance of members of the family Enterobacteriaceae in DA-DTT versus DA-U

	DA-DTT (n = 43) vs. DA-U (n = 112)				
Taxonomy	Average relative abundance % DA-DTT^a^	Average relative abundance % DA-U^a^	Fold Change (calculated from the GLM)^b^	*P*-value	FDR *P*-value
c_Gammaproteobacteria, o_Enterobacteriales, f_Enterobacteriaceae	19.193	6.068	5.46	**5.13E-4**	**1.47E-3**
f_Enterobacteriaceae, g_unknown	14.984	5.227	17.00	**2.72E-8**	**1.21E-7**
f_Enterobacteriaceae, g_Klebsiella	3.812	0.784	24.10	**7.13E-7**	**2.73E-6**
f_Enterobacteriaceae, g_Providencia	0.224	0.00025	13.57	**< 0.0001**	**< 0.0001**
f_Enterobacteriaceae, g_Morganella	0.018	0.006	36.71	**1.18E-9**	**5.81E-9**
f_Enterobacteriaceae, g_Salmonella	0.006	0.001	3.71	**0.01**	**0.02**

*P*-value< 0.05 and FDR *P*-value< 0.05 are shown in bold. ^a^The relative abundances were calculated from the original counts (number of sequences in the OTU table). ^b^Fold changes were calculated from the GLM, which corrects for differences in library size between the samples and the effects of confounding factors. It is therefore not possible to derive these fold changes from the original counts (number of sequences in the OTU table) by simple algebraic calculations

The increase in detected relative abundances of *Alphaproteobacteria* and *Deltaproteobacteria* in DA-DTT (Table 3) was driven by increases in detection of the order *Rhizobiales* (FC = 19.03, FDR *P* = 4.33E-13), and of sulfur-producing bacteria from the orders *Desulfobacterales* (FC = 42.61, FDR *P* < 0.0001) and *Desulfovibrionales* (FC = 6.41, FDR *P* = 6.71E-3), respectively.

#### Pretreatment with DTT does not affect microbial diversity in DA

**Fig. 5:**
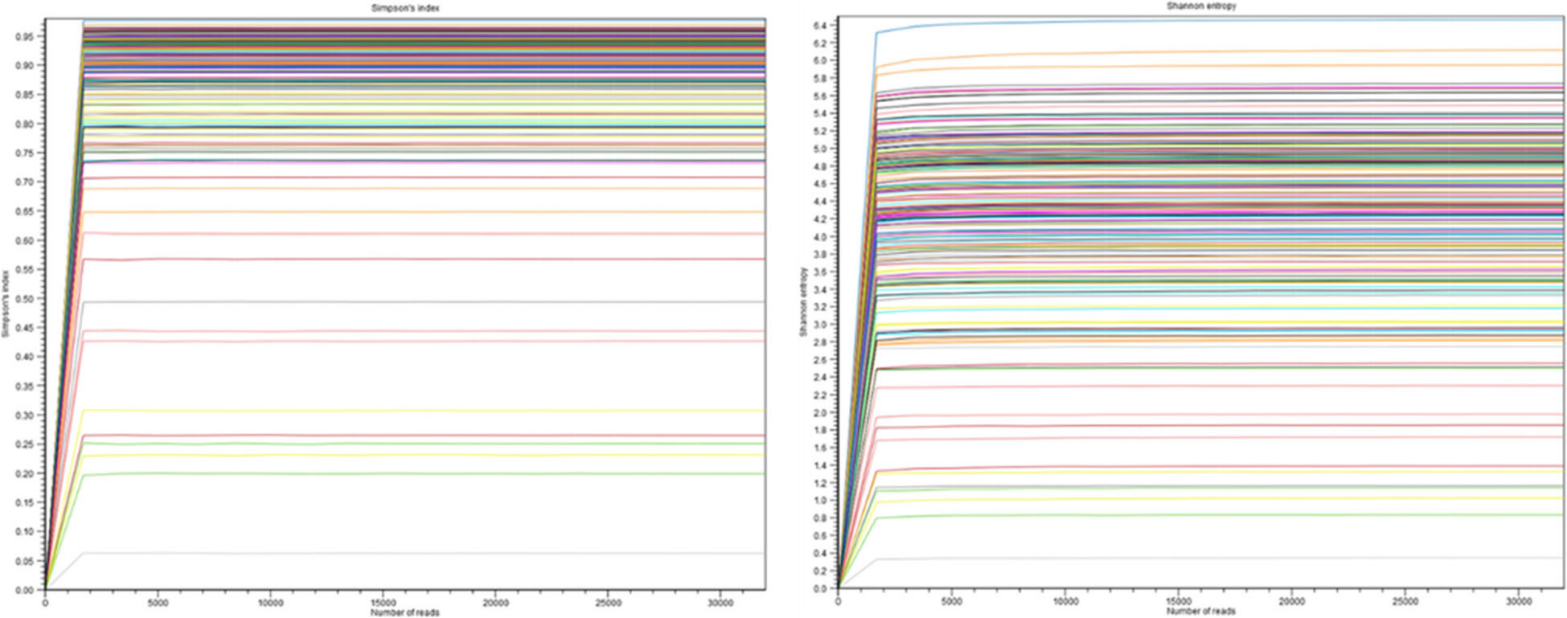
Alpha diversity rarefaction curves for DA-DTT (*N* = 112) and DA-U (*N* = 43) samples. Samples were rarefied to the least numbers of sequences obtained

**Fig. 6:**
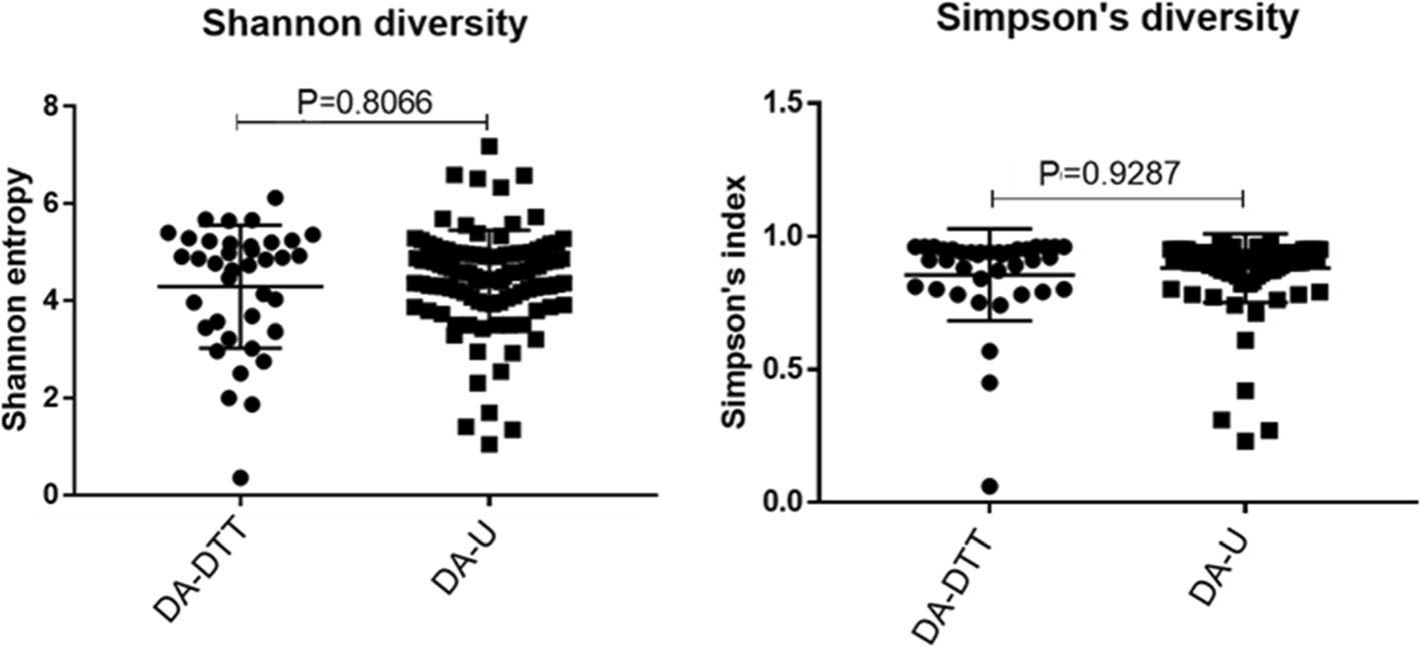
Alpha diversity indices of DA samples pre-treated with DTT (DA-DTT, *N* = 112) and untreated DA (DA-U, *N* = 43). Left: Shannon entropy diversity for DA-DTT and DA-U samples. Right: Simpson’s index diversity for DA-DTT and DA-U samples

**Fig. 7:**
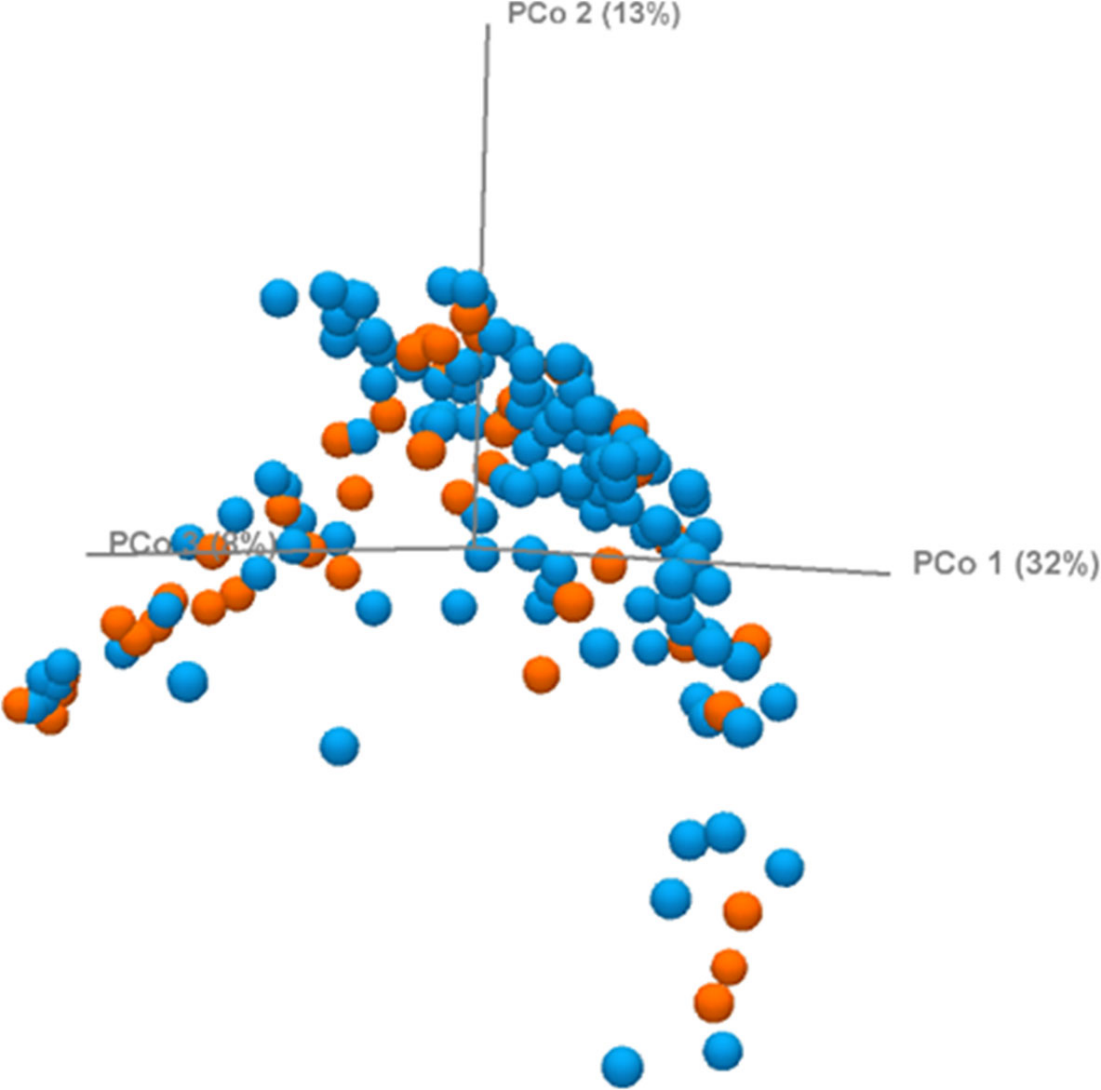
Beta diversity of DA-U and DA-DTT based on the weighted UniFrac metric. Principal Coordinates Analysis plot of binary and abundance-weighted Unifrac distances between DA-DTT (shown in orange, *N* = 43) and DA-U (shown in blue, *N* = 112)

Sample rarefaction curves showed a similar pattern, which verified that most of the species present in each sample from DA-DTT and DA-U groups were observed (Fig. 5) [11]. DA-DTT exhibited the same alpha diversity as DA-U, as demonstrated by Simpson’s index (*P* = 0.9287) and Shannon entropy (*P* = 0.8066) (Fig. 6). Beta diversity of the DA-U and DA-DTT microbiome was analyzed based on the weighted UniFrac metric. Principal Coordinate Analysis plot showed no clustering of the DA-DTT (*n* = 43) and DA-U groups (*n* = 112) (see Fig. 7).

## Discussion

In this paper, we develop and validate a novel methodological approach based on the use of the reducing agent dithiothreitol (DTT) that resolves issues related to low microbial biomass from luminal duodenal aspirates (DA). The use of DTT clearly increases the number of bacteria detected on culture plates, and also increases DNA yields and the concentration of V3/V4 libraries for sequencing, which in turn results in important differences in the microbial populations detected in DA.

Given the central role of the small intestine in the processes of digestion and nutrient absorption, accurate characterization of the human small intestinal microbiome is an important future consideration. The small intestine is not as heavily colonized as the large intestine, ranging under healthy conditions from 10^3^ to 10^4^ bacteria per mL of intestinal content in the duodenum and jejunum to 10^8^ bacteria per mL in the ileum, compared to 10^11^ bacteria per gram of wet stool in the colon [12, 13]. In addition to having low bacterial biomass, duodenal luminal contents are viscous due to the mucus layer present in the small intestine [14, 15], and require special handling during sample collection and processing prior to culture and DNA extraction, in order to increase the likelihood of the assessment of all microbial communities, including those associated with the mucus layer.

In the assessment of the microbiome, it is essential to accurately and completely assess the microbial components in a sample. Although standards have been set for the assessment of the stool microbiome, these standards have not been assessed for small intestinal fluid assessment. Mucous in general is a viscous fluid that can trap bacteria in its matrix and previous studies performed with sputum samples have shown that treating this viscosity has an impact on the microbial assessment [5, 16]. However, until now, no studies have investigated the impact on microbial assessment and DNA recovery in aspirates collected from small bowel. There are agents known to safely and effectively improve microbial assessment and DNA yield in viscous samples, and one such agent is DTT, which can reduce the disulfide bonds between mucin subunits.

In this study we established a methodology to improve microbial DNA recovery from small bowel aspirates, which includes different sample processing steps when compared to conventionally published methods for extracting DNA for microbiome assessment of gut materials such as stool [17–19]. The concentrations of DNAs extracted from DA ranged from very low levels to up to 70 ng/ml when samples were pretreated with DTT, more than 3-fold higher than those from samples which were not pretreated with the reducing agent. Initial DNA concentrations exhibited a higher positive correlation with those of the final V3/V4 libraries for DTT-pretreated DA compared to non-pretreated DA, which may indicate a specific increase in the isolation of bacterial DNAs. The use of a fixed initial DNA concentration during the preparation of sequencing libraries from DA should be carefully analyzed, and the addition of DTT during sample processing and for removal of the All Protect reagent is highly recommended as this increases the initial yield of microbial DNA prior sequencing library preparation.

In addition to increases in DNA yields and library concentrations, DA processed with DTT prior to microbial culture exhibited higher numbers of bacterial colonies on blood agar plates incubated under anaerobic conditions. The relative abundance of specific obligate and facultative anaerobes detected in DA-DTT was increased compared to DA-U samples, which may reflect the increased detection of microbes associated with the mucus layer. For example, the relative abundance of the genus *Enterococcus* (phylum *Firmicutes*) detected in DA-DTT was significantly increased. This genus comprises over 50 Gram-positive facultative anaerobic lactic acid cocci species isolated from numerous environments, including the human GI tract [20]. *Enterococcus* species constitute up to 1% of the gut microbiota, and most species can grow on blood agar plates under anaerobic conditions. The relative abundance of the genus *Clostridium* (phylum *Firmicutes*), which is comprised of obligate anaerobes and some aerotolerant species, detected in DA-DTT samples was also increased.

The relative abundance of the genus *Bacteroides*, comprised of Gram-negative obligately anaerobic bacilli, detected in DA-DTT was also significantly increased compared to DA-U samples. Species from this genus can grow on blood agar and are well-adapted to the gastrointestinal tracts of mammals, including humans [21, 22]. The human large intestine is densely colonized with species from the genus *Bacteroides* (phylum *Bacteroidetes*) [23], many of which perform essential metabolic functions for the host, including the metabolism of proteins and complex sugars. In contrast, the small intestine is not as heavily colonized by members of the phylum *Bacteroidetes*, which comprised less than 4% of the total microbes detected in DA-U. With the addition of the reducing agent DTT, which breaks the disulfide bonds linking mucin subunits in mucus prior to microbial culture and DNA extraction, the relative abundance of *Bacteroidetes* detected in DA increased significantly from 4 to 7%, indicating a possible role for species from this phylum in mucus metabolism. Mucus is a dynamic matrix, consisting of mucin glycoproteins secreted by intestinal goblet cells, which lubricates the transit of intestinal contents, amongst other functions. Mucus glycoproteins can be used as a carbon source by many asaccharolytic microorganisms, and the low oxygen levels at atmospheric pressures allow the colonization and growth of anaerobes in mucus [24, 25].

The phylum *Proteobacteria* also includes aerotolerant asaccharolytic microorganisms that require proteinaceous substrates as carbon and energy sources, such as *Campylobacter* [26]*,* as well as facultative anaerobes from the family Enterobacteriaceae included in the “Mucosally Associated Consortium” in the colon described by Albenberg et al. [24]. Pretreatment of DA with DTT increases the detected relative abundance of many *Enterobacteriaceae* members, including the clinically important genera *Klebsiella, Providencia* and *Salmonella* as well as unknown members. *Providencia* and *Salmonella* include motile species that can adhere to mucus and epithelial cells and actively invade the host epithelium [27–29]. The relative abundance of the genus *Pseudomonas*, detected in DA-DTT was also increased compared to non-pretreated DA. Members of this genus, including the most studied species *P. aeruginosa,* are also motile and can be part of the normal human microflora, but are also important clinically as they are known to cause hospital-acquired infections such as pneumonia and urinary tract infections [30].

A limitation of this study was that DTT treatment vs. non-treatment could not be tested in different portions of the same samples. This was partly due to the small sizes of the individual samples, and partly due to the fact that their viscosity made it impossible to divide them evenly. We have attempted to compensate for this through the number of samples tested.

Surprisingly, the changes in several microbial taxa in DA samples after the addition of DTT did not affect the overall microbial diversity. These findings further suggest that the addition of the reducing agent DTT improves microbial assessment and DNA recovery without causing a dramatic change in the microbial balance in the aspirate samples.

## Conclusions

This study validates methodology to optimize yield for culture, and for DNA extraction for analysis of the small bowel microbiome. Culture totals, microbial DNA and microbiome analysis demonstrate marked differences with this new technique. This suggests that conventional techniques for DNA isolation provide an incomplete picture of the microbial environment in the small bowel. Thus, this new technique appears ideal for small bowel microbiome assessment.

## Methods

The REIMAGINE study is a large-scale study designed to examine the relationship between the small bowel microbiome in human health and disease. In brief, the study involves collecting data in consecutive patients undergoing routine upper gastrointestinal endoscopy (esophagogastroduodenoscopy). After a battery of questionnaires for comprehensive collection of health information, and obtaining serum and genetic samples, the endoscopy entailed the collection of small bowel aspirates and two mucosal biopsies. The first phase of this study was to develop, validate and optimize small intestinal aspiration techniques, and microbial sample acquisition.

### Study subjects

Male and female subjects aged 18–85 undergoing esophagogastroduodenoscopy (EGD) without colon preparation for standard of care purposes were prospectively recruited for this study. Potential participants were identified by study staff and their eligibility was verified by co-investigators or the PI. Although there are no exclusion criteria for the REIMAGINE study, small bowel biopsies are not collected from subjects with bleeding disorders or advanced cirrhosis of the liver with coagulopathy and intestinal varices where the international normalized ratio (INR) was greater than 1.5, in order to minimize the risk of bleeding from the biopsy site. The REIMAGINE study protocol was approved by the Institutional Review Board at Cedars-Sinai Medical Center, and all subjects provided informed written consent prior to participating in the study. Samples from subjects taking antibiotics were not included in the present study.

### Study procedures

#### Small intestinal sample procurement

During EGD, samples of luminal fluid (~ 2 ml) were obtained from the duodenum using a custom sterile aspiration catheter (Hobbs Medical, Inc.). The custom catheter consisted of a newly designed double lumen sterile catheter, with the inner lumen maintaining sterility during insertion by applying sterile bone wax into the open tip of the external catheter. During endoscopy, the endoscopist is instructed to immediately enter the duodenum and insert the aspiration catheter. The inner catheter then dislodges the bone wax, exposing the sterile inner catheter. This inner catheter is used to aspirate duodenal fluid through lasered side holes to acquire a volume of 2 mL. These precautions eliminated the risk of oral and gastric contamination.

#### Aspirate processing

Immediately after aspiration, samples from all duodenal aspirates were cultured on MacConkey agar (Becton Dickinson, Franklin Lakes, NJ, EUA) and blood agar (Becton Dickinson) for determination of the number of colony-forming units (CFU) per mL of aspirate. To assess the effect of viscosity on culture, a subset of aspirates were not pretreated and simply cultured (the DA-U group for “untreated”) and another subset were first pretreated with the reducing agent Dithiothreitol (DTT) (Sputolysin® Reagent, Cat. 560,000-1SET, EMD Millipore Corp, Billerica, MA, USA) (the DA-DTT group) (see Fig. 1).

For DA-DTT samples, 1x DTT (6.5 mM dithiothreitol in 100 mM phosphate buffer, pH 7.0) was added to an aliquot of the DA in a 1:1 ratio and the resulting mixture was vortexed until the sample was liquified (typically 30 s). 100 μL of the liquified mixture was serially diluted with 900 μL sterile 1x PBS and samples of the 1:10 and 1:100 dilutions were plated in duplicate on MacConkey agar under aerobic conditions for the quantitation of Gram-negative bacilli, and on blood agar under anaerobic conditions for the quantitation of total anaerobes. For DA-U samples, 100 μL of DA was diluted directly with 900 μL sterile 1x PBS and samples of the 1:10 and 1:100 dilutions were plated as described above. All plates were incubated at 37 °C for 16–18 h, after which colonies were electronically counted using the Scan 500 (Interscience, Paris, France). As a negative control, 100 μL of 1x DTT was also cultured aerobically on MacConkey agar and anaerobically on blood agar. All dilution factors were taken into account for final determination of microbial burden.

After aliquots for microbial culture were taken, remaining DA-U and DA-DTT samples were centrifuged at high speed (17,136 x g) for 10 min and the supernatant was carefully removed and stored at − 80 °C for future metabolomic analyses. 500 μL of sterile All Protect reagent (Qiagen, Hilden, Germany) was added to each pellet for stabilization of DNA, RNA and proteins, and the pellets were stored at − 80 °C prior to DNA isolation and analysis of the DA microbiome.

#### DNA extraction and quantification from aspirates

DA-U and DA-DTT samples were thawed on ice and 1x DTT was added in a 1:1 ratio, after which the samples were vortexed until the All Protect reagent was fully liquefied (around 30 s). DNA extraction was then performed for both groups using the MagAttract PowerSoil DNA KF Kit (Qiagen, cat. No. 27000–4-KF) with some modifications. DNA extraction was also performed on negative control samples (1x DTT) as a control.

The lysis step was carried out by adding garnet beads (Qiagen, cat. No. 13123–50) and 746 μL PowerBead Solution to each pellet-containing tube, followed by 4 μL RNase A and 60 μL SL Solution (Lysis buffer) in this specific order. Tubes were sealed with parafilm, vortexed horizontally for 15 min, and then centrifuged for 6 min at 4500 x g. The supernatants were transferred to new tubes containing 450 μL IR Solution, vortexed for 3 s, incubated at 4 °C for 10 min, and then centrifuged for 6 min at 4500 x g. The supernatants were transferred to new tubes and centrifuged for a further 6 min at 4500 x g. 450 μL of the resulting supernatants were added to deep 96-well KingFisher plates containing magnetic beads and DNA extraction was performed using the KingFisher Duo (Thermo Fisher Scientific, Waltham, MA, USA). The final DNA volume was 100 μL. DNAs were then quantified using Qubit ds DNA BR Assay kits (Invitrogen by Thermo Fisher Scientific, Waltham, MA, USA) on a Qubit 4 Fluorometer (Invitrogen).

#### Library preparation and 16S rRNA gene sequencing

16S library preparation for DNAs from all groups was performed according to the Illumina (Illumina, San Diego, CA, USA) protocol https://support.illumina.com/documents/documentation/chemistry_documentation/16s/16s-metagenomic-library-prep-guide-15044223-b.pdf, with some modifications. The V3 and V4 regions were amplified using the gene-specific primers S-D-Bact-0341-b-S-17 and S-D-Bact-0785-a-A-21 published and validated by Klindworth et al. [31]. The primers were modified in accordance with the protocol by adding the Illumina sequencing adapters to each one.

The full-length primer sequences used were:

16S amplicon PCR forward primer: 5’TCGTCGGCAGCGTCAGATGTGTATAAGAGACAGCCTACGGGNGGCWGCAG

16S amplicon PCR reverse primer: 5’GTCTCGTGGGCTCGGAGATGTGTATAAGAGACAGGACTACVHGGGTATCTAATCC

The 16S library preparation protocol was modified as follows: 5 μL of DNA was added to a Master Mix (0.5 μL of 10 μM 16S Amplicon PCR Forward primer, 0.5 μL of 10 μM 16S Amplicon PCR Reverse primer, 12.5 μL 2x KAPA HiFi HotStart ReadyMix and 6.5 μL of molecular grade PCR H_2_O) and the PCR was performed as follows:

1. Initial denaturation step at 95 °C for 3 min
2. 27 cycles of: 95 °C for 30 s, 55 °C for 30 s and 72 °C for 30 s
3. 72 °C for 5 min
4. Hold at 4 °C

An optimized Clean-Up step was performed with Agencourt AMPure XP beads using the modifications proposed by Quail et al. [32]. After adding the beads to each Amplicon PCR 96-well plate on the magnetic stand, samples were incubated for five minutes followed by two wash steps with 80% ethanol. The beads were air dried for five minutes. After removing the plate from magnetic stand, beads were incubated with EB Buffer (Qiagen) for five minutes to elute the DNA. The plate was placed back on the magnetic stand and after 2–3 min the supernatant was transferred to an empty clean well, preventing the transfer of the beads with the supernatant.

Five μL of the final Amplicon PCR product was used for the Index PCR, which was performed using the Nextera XT Index kit and 2x KAPA HiFi HotStart ReadyMix, following the Illumina protocol for 8 cycles. After a second modified Clean-Up step, the final product was quantified using Qubit ds DNA BR Assay kits and Qubit 1X dsDNA HS Assay kits on a Qubit 4 Fluorometer and analyzed using Agilent DNA 1000 chips (Agilent Technologies, Santa Clara, CA) and Agilent HS DNA chips (Agilent) on an Agilent 2100 Bioanalyzer System.

### 16S rRNA gene sequencing and analysis

The V3 and V4 libraries prepared using DNAs from DA-DTT and DA-U groups were sequenced using a MiSeq Reagent Kit v3 (600-cycles) on a MiSeq System (Illumina, San Diego, California). 2 × 301 cycles paired-end sequencing was performed according to manufacturer’s protocol and 5% Phix (Illumina) was added to each library pool.

Operational Taxonomic Unit (OTU) clustering and taxonomic analyses were performed using CLC Genomics Workbench v. 10.1.1 and CLC Microbial Genomics Module v. 2.5 (Qiagen). Sequences were first trimmed to remove 13 bases at the 5′ terminal position and merged considering the alignment scores as follows: mismatch cost of 2, gap cost of 2, zero maximum unaligned end mismatches and minimum score of 30. After merging, sequences were clustered into OTUs at 97% sequence similarity level using the Amplicon-Based OTU clustering tool. The creation of new OTUs was allowed considering 97% taxonomic similarity. The most abundant sequences were selected as representative of each cluster, and then assigned to a taxonomy level using CLC Microbial Genomics default values and the Greengenes Database 2013 release. Alpha diversity indexes (Chao1, Simpson and Shannon) were calculated using the Abundance Analysis tool. The weighted Unifrac metric was used to calculate inter-sample diversity (beta diversity).

#### Statistical analysis

Multiple comparisons and statistical analyses were performed using CLC Genomics Workbench v. 10.1.1 and CLC Microbial Genomics Module v. 2.5 (Qiagen). A Negative Binomial Generalized Linear Model (GLM) model was used to obtain maximum likelihood estimates for an OTU’s log-fold change between two conditions, and the Wald test was used to determine significance, as part of the CLC package available at https://www.qiagenbioinformatics.com/products/clc-genomics-workbench/. False Discovery Rate (FDR) was performed to correct *P*-values. Fold changes are calculated from the GLM, which corrects for differences in library size between the samples and the effects of confounding factors. Again, these calculations were performed using the CLC package. It is therefore not possible to derive these fold changes from the original counts by simple algebraic calculations. Two-tailed Spearman r correlations, Mann-Whitney tests and graph construction were performed using GraphPad Prism 7.02 (GraphPad Software, La Jolla, CA, USA). For statistical analysis purposes only, no growth on blood agar and MacConkey agar (CFU/ml = 0) was assigned as 1 CFU/ml.

## Supplementary information

**Additional file 1. **Title of data: Culture data, sequenced samples information and OTU table. Description of the data: the culture data section (sheet 1) shows the number of bacterial colonies observed on MacConkey agar (incubated aerobically) and blood agar (incubated anaerobically) for all DA-DTT (*N* = 101) and DA-U (*N* = 127) samples. Data are presented as CFU/mL of duodenal aspirate. The sequenced samples information section (sheet 2) provides data for the sequenced DA-DTT (N = 43) and DA-U (N = 112) samples, including the number of bacterial colonies observed on MacConkey agar and blood agar (in CFU/mL of duodenal aspirate), initial DNA concentration, library concentration and library size (number of sequences). The OTU table section (sheet 3) contains the OTU table generated from the sequenced DA-DTT (N = 43) and DA-U (N = 112) samples. The table provides the OTU numbers, taxonomic classification, number of reads for each OTU and the OTU sequence.

**Additional file 2.** Title of data: V3-V4 16S rRNA library size of DA-DTT and DA-U samples. Description of the data: library sizes (number of sequences in the OTU table) for DA-DTT and DA-U samples. The table provides the library size mean, standard deviation, standard error of the mean, median, 25 and 75% percentiles.

## Data Availability

The datasets generated during the current study are available in the National Center for Biotechnology Information (NCBI) BioProject Repository https://www.ncbi.nlm.nih.gov/bioproject under BioProject ID PRJNA520899.

## Abbreviations

CFU: Colony-forming units
DA: Duodenal aspirates
DA-DTT: Duodenal aspirates pretreated with dithiothreitol
DA-U: Duodenal aspirates untreated
DNA: Deoxyribonucleic acid
DTT: Dithiothreitol
EGD: Esophagogastroduodenoscopy
FC: Fold change
FDR: False discovery rate
GI: Gastrointestinal
GLM: Generalized linear model
INR: International normalized ratio
Med: Median
OTU: Operational taxonomic unit
PCR: Polymerase chain reaction
REIMAGINE study: Revealing the Entire Intestinal Microbiota and its Associations with the Genetic, Immunologic, and Neuroendocrine Ecosystem study
rRNA: ribosomal ribonucleic acid
