# Supplementary material for: Optimizing microbiome sequencing for small intestinal aspirates: validation of novel techniques through the REIMAGINE study
Source: BMC Microbiol. 2019 Nov 1;19:239. doi: 10.1186/s12866-019-1617-1 (PMC6824053; doi:10.1186/s12866-019-1617-1)
Supplement: Supplementary file 2 — Additional file 2. Title of data: V3-V4 16S rRNA library size of DA-DTT and DA-U samples. Description of the data: library sizes (number of sequences in the OTU table) for DA-DTT and DA-U samples. The table provides the library size mean, standard deviation, standard error of the mean, median, 25 and 75% percentiles. [file 12866_2019_1617_MOESM2_ESM.pdf]

Additional file 1. V3-V4 16S rRNA library size of DA-DTT and DA-U samples

	DA - All subjects (n=155)	
Library size (sequences)	Non-pretreated (n=112)	DTT-pretreated (n=43)
Mean	236,525	174,525
Standard Deviation	157,694	92,366
Standard Error of Mean	14,901	14,086
Median	199,936	169,737
25% Percentile	118,973	101,216
75% Percentile	329,521	199,936
